# The Effects of Digital Marketing of Unhealthy Commodities on Young People: A Systematic Review

**DOI:** 10.3390/nu10020148

**Published:** 2018-01-29

**Authors:** Limin Buchanan, Bridget Kelly, Heather Yeatman, Kishan Kariippanon

**Affiliations:** 1 School of Health and Society, Early Start, Faculty of Social Sciences, University of Wollongong, Northfields Ave, Wollongong, NSW 2522, Australia; bkelly@uow.edu.au; 2 School of Health and Society, Faculty of Social Sciences, University of Wollongong, Northfields Ave, Wollongong, NSW 2522, Australia; hyeatman@uow.edu.au (H.Y.); kishan@uow.edu.au (K.K.)

**Keywords:** digital marketing, online marketing, unhealthy commodities, young people, consumption behaviors, systematic review

## Abstract

The marketing of unhealthy commodities through traditional media is known to impact consumers’ product attitudes and behaviors. Less is known about the impacts of digital marketing (online promotional activities), especially among young people who have a strong online presence. This review systematically assesses the relationship between digital marketing and young people’s attitudes and behaviors towards unhealthy commodities. Literature was identified in June 2017 by searches in six electronic databases. Primary studies (both qualitative and quantitative) that examined the effect of digital marketing of unhealthy food or beverages, alcohol and tobacco products on young people’s (12 to 30 years) attitudes, intended and actual consumption were reviewed. 28 relevant studies were identified. Significant detrimental effects of digital marketing on the intended use and actual consumption of unhealthy commodities were revealed in the majority of the included studies. Findings from the qualitative studies were summarized and these findings provided insights on how digital marketing exerts effects on young people. One of the key findings was that marketers used peer-to-peer transmission of messages on social networking sites (e.g., friends’ likes and comments on Facebook) to blur the boundary between marketing contents and online peer activities. Digital marketing of unhealthy commodities is associated with young people’s use and beliefs of these products. The effects of digital marketing varied between product types and peer endorsed marketing (earned media) may exert greater negative impacts than owned or paid media marketing.

## 1. Introduction

Non-communicable diseases (NCDs) are the leading causes of death and disability across many nations [1]. Major modifiable risk factors for NCDs include the consumption of unhealthy commodities such as ultra-processed, energy-dense nutrient-poor (EDNP) food and beverages, tobacco and alcohol [2]. Commercial marketing of these unhealthy commodities propagates their use, where this marketing is ubiquitous, repetitive and reinforced across media platforms. Evidence of the effects on behaviors of marketing various unhealthy commodities, as summarized by previous reviews, has found a remarkable degree of consistency regarding the widespread and detrimental effects of these marketing efforts [3,4,5,6,7,8,9].

Most of the available literature that explores the effects of marketing unhealthy commodities focuses on traditional broadcast media, namely television [4,6,7]. Although television remains the most utilized promotional channel, evidence suggests that its dominance is waning [10]. Detailed expenditure data for unhealthy commodities marketing are not publicly accessible but what is available indicates that the manufacturing food industry is shifting their marketing budgets from traditional media to digital media. In the USA, spending on children-directed television marketing has experienced a decline of 20% while spending on digital media marketing rose by 50% between 2006 and 2009 [10].

Digital marketing encompasses any promotional activities undertaken through websites, social networking sites (SNS), emails, mobile phone texts, applications (apps) and online games [11]. This form of marketing is well-regarded in the literature within marketing and advertising research fields for its ubiquity, interactivity and 24/7 availability [12]. There is a suggestion that digital marketing is even more impactful than traditional marketing due to its characteristic of peer endorsement and lack of explicit advertising cues presented in some forms of digital media [11], for instance, ‘seeding’ a message on SNS and transmitting this through online communities.

The vast majority of research that has been undertaken to understand the effects of commercial marketing of unhealthy commodities has focused on young children [4,6,7], whereas research on older adolescents and adult populations are relatively limited [3,9,13]. One review systematically assessed the evidence of experimental intervention studies of food and beverages promotion to adults (16 years and above) but could not draw a conclusive outcome from the sparse studies conducted on adults [13]. In two systematic reviews that summarized evidence from prospective cohort studies on alcohol promoted to adolescents and young adults through traditional media, marketing was linked to early onset of drinking [3] and current alcohol consumption [9]. Public health researchers have called for greater policy and research attention on the effect of marketing on behaviors and for this to be extended to adolescents and young adults due to the rising overweight and obesity rates [14,15] and the lack of regulations in restricting unhealthy commodities, particularly EDNP food and beverages, towards these age groups [15].

Adolescents and young adults have a strong online presence and growing purchase power and are viewed by digital marketers as a lucrative market segment [15,16]. Yet only a few studies have assessed the use of digital media to promote unhealthy commodities to these age groups by the food and beverages [15], tobacco [17] and alcohol [18] industries and these studies have only looked at one unhealthy commodity in isolation due to different research interests. There are parallels in the marketing techniques used between these different product categories and so collective review evidence of effects may be useful. The aims of this review were to: (i) systematically assess the findings from empirical studies that evaluate the association between digital marketing and young people’s attitudes and behaviors towards unhealthy commodities; and (ii) collate findings to provide an overview of how this novel form of marketing exerts its influences on young people. This review will be valuable for the public health community and policymakers to assist their understandings of the contributions of digital marketing to the use of unhealthy commodities and the need for policy interventions, for example regulatory interventions to restrict this marketing and programs to counter the effects of unhealthy digital marketing.

## 2. Materials and Methods

This review is reported consistent with the PRISMA (preferred reporting items for systematic reviews and meta-analyses) guidelines [19]. The protocol was registered with the PROSPERO International Prospective Register of Systematic Review before commencing of data extraction [20] (see protocol registration number: CRD42017076682). A systematic literature search was conducted in June 2017 on databases including Business Source Complete, Emerald Insight, ProQuest Central, PsycINFO, Scopus and Web of Science for articles published between 1990 and 2017 and whose title, abstract and keywords matched the following Boolean search strings: (market* OR advert* OR promot*) AND (online OR internet OR web OR “social media” OR “social network” OR “new media” OR “online game” OR advergam*) AND (“young people” OR “young adults” OR “young generation” OR “university students” OR “college students” OR adolescents OR teenagers OR youths) AND NOT (child*) AND (food OR beverage OR drink OR soda OR cola OR alcohol OR tobacco OR cigarette). The full record of search strings on Scopus is shown in Table 1. Additional searches for suitable studies were conducted on Google Scholar, websites and grey literature sources. Reference lists of the identified articles and key reviews were hand-searched for further studies.

Studies were included if they met the following criteria: (1) Primary studies published in peer-reviewed journals or on relevant websites; not reviews or commentaries; (2) Study participants were aged 12 to 30 years; studies with a sample of broader age range were only included if the target age group was analyzed separately. Australian Bureau of Statistics (ABS) identified adolescents and young adults aged 15–34 years as the biggest Internet users (98%) followed by adolescents aged under 15 years (97%) [21]. The age range of 12 to 30 years was selected since many studies have used 12 years as a starting year for adolescents and 30 years as a cut-off for young adults; (3) Study factors included any marketing or promotion of unhealthy commodities including food and beverages, tobacco and alcohol generated by the product industry using online platforms such as Internet, websites, social network sites (SNS), online games and emails. Studies that examined the marketing impact of digital media as well as traditional media were included if the effects of digital marketing were analyzed separately. Studies that examined the user-generated online contents were excluded; (4) Indicators of outcome included psychological measures such as perception and attitudes, purchase and consumption intentions, purchase and consumption behaviors (for the simplicity, hereby collectively referred to as “attitudes and behaviors”); and (5) Studies based on content analyses were excluded as outcome variables were often not examined in this type of study.

Title and abstract of the identified references were pre-screened for relevance by the lead reviewer (L.B.). Two independent reviewers (L.B., K.K.) then assessed the full-text articles in detail based on the exclusion criteria. When there were discrepancies between the two reviewers, a third reviewer (B.K.) was consulted. A consensus was then reached through discussion of evaluations. Data of the included studies were extracted and recorded in a tabulated summary by the lead reviewer. Details recorded in the template included date, location, participants’ demographics, sample size, study aims, study designs, study factors, outcome measures, results and control variables. Overall association between the study factors and outcome measures were determined and categorized into: significant detrimental association (e.g., increased exposure enhanced unhealthy commodities use); significant beneficial association (e.g., increased exposure reduced unhealthy commodities use); association cannot be determined; or inconsistent association (e.g., a mixture of detrimental, beneficial, or no association). The second reviewer (K.K.) verified the extractions.

Due to the heterogeneity of the study designs, study factors and outcome measures of the included studies (see Section 3), meta-analysis was deemed inappropriate and qualitative narrative synthesis was used to combine the overall findings of the reviewed studies. Quality appraisals were conducted by using the NIH (National Heart, Lung and Blood Institute, Bethesda, MD, USA) (cross-sectional, longitudinal and controlled intervention studies) or CASP (Critical Appraisal Skills Program) (qualitative studies) assessment tools [22,23,24]. Each tool can be generally divided into four domains: study setup, sample selection, assessment and data analysis. Each domain was rated good, fair or poor. Studies included in the review were appraised by the lead reviewer in consultation with the second and third reviewers.

## 3. Results

**Figure 1:**
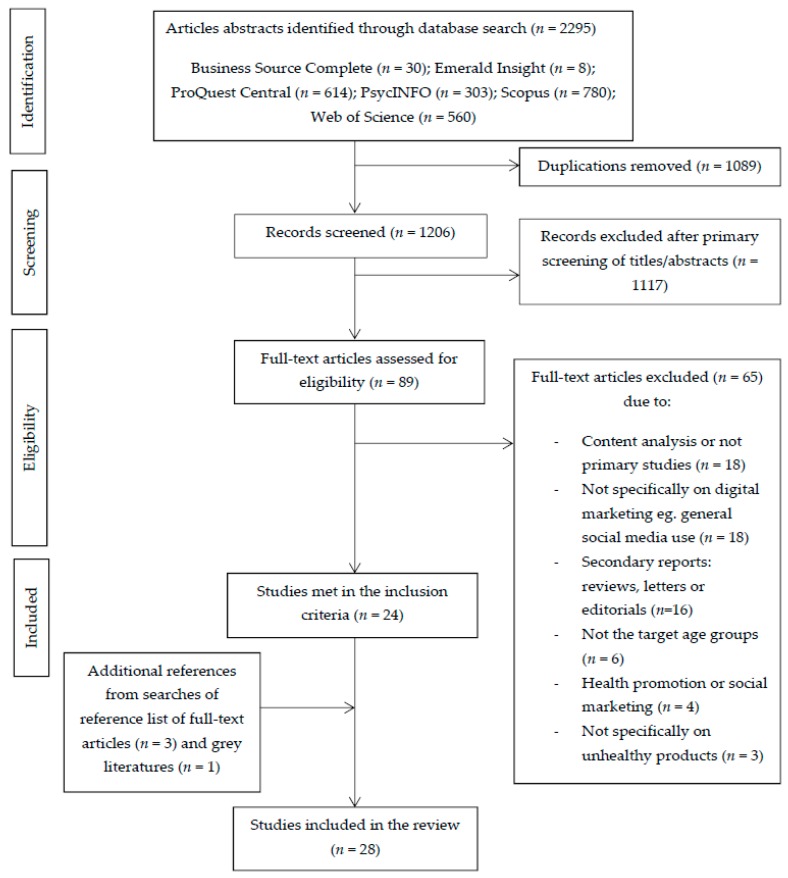
Flow chart of systematic review literature search.

Database searches identified 2295 records, with 1206 records remaining after duplicates were removed. The primary screening excluded 1117 records; the remaining 89 full-text articles were assessed in detail and 24 met the inclusion criteria. An additional four studies were identified through the reference lists of the identified articles and grey literature sources. A total of 28 articles were included in this review (Figure 1).

The included 28 studies were published between 2001 and 2017 (Table 2 and Table 3). The majority of the studies were conducted in economically developed countries (*n* = 24): Australia (*n* = 9) [25,26,27,28,29,30,31,32,33], USA (*n* = 5) [34,35,36,37,38], UK (*n* = 6) [39,40,41,42,43,44] and New Zealand (*n* = 3) [45,46,47]. One study included data collected from four countries—Germany, Italy, the Netherlands and Poland [48]. One study was conducted in each of Argentina [49], Brazil [50], Egypt [51] and Fiji [52]. The included studies reported data on 7 to 22,007 participants aged 12 to 30 years. Of these, six studies drew findings from state-wide or national samples [26,27,31,32,38,48]. The majority of the included studies examined marketing effects of alcohol (*n* = 18) [26,28,29,30,33,34,36,39,40,42,43,44,45,46,47,48,50,52], some were of tobacco or e-cigarettes (*n* = 7) [27,31,35,37,38,41,49] and a small number were on EDNP food or beverages (*n* = 3) [25,32,51].

Eleven of the included studies investigated the effects of unhealthy commodities marketed through digital media as well as traditional media [30,31,32,35,37,38,39,40,41,45,50]. Seventeen studies specifically examined marketing impacts of unhealthy commodities through digital communications [25,26,27,28,29,33,34,36,42,43,44,46,47,48,49,51,52]. Where percentage data were available, the reported prevalence of exposure to, or engagement with, digital marketing ranged from 5% to 88%; only four studies reported greater than 50% rates [26,28,39,48]. The outcomes of interest of the included studies were classified into three categories, with some studies measuring more than one outcome variable: attitudes (i.e. beliefs, perceptions of brands or products) (*n* = 10) [25,33,37,42,43,44,46,47,51], intended use (*n* = 9) [25,26,27,30,34,36,40,45,48] and current use (*n* = 17) [26,27,28,29,30,31,32,35,36,38,39,40,41,45,48,49,50] of unhealthy commodities.

It was difficult to quantify the volume of studies that found a detrimental association between digital marketing and attitudes or behaviors given that most of the included studies examined more than one form of digital marketing or outcome measure and mixed findings were found in some. Inconsistency of the direction of associations were found in eight studies [27,29,30,34,36,37,45,49] but all studies found at least one significant detrimental association between the examined study factors and outcome measures. Among the rest of the studies, nine studies found consistent significant detrimental associations [25,26,28,32,35,38,40,41,46], one study found consistent significant beneficial associations [31] and associations could not be determined in three studies [32,41,50] since regression analyses were not performed although descriptive data (e.g., percentage of exposure rate) were used to suggest the relationship (Table 2). The effects of digital marketing are therefore discussed by three outcomes of interest categories in the following sections.

Detrimental effects on young people’s attitudes towards unhealthy commodities or brands were demonstrated in two quantitative studies [25,37] and supported by five qualitative studies [33,42,43,46,47] (Table 2 and Table 3). One study found that participants’ interests in energy drinks was enhanced by the social corporate responsibility efforts and masculine images created by the energy drinks brands after being exposed to the brands’ websites and SNS [25]. Another study linked e-cigarette advertisements on the Internet to lower perceived harm and greater acceptability of these products among university students [37]. Association in one study cannot be determined given that only descriptive analyses were conducted. However, this study reported that digital alcohol marketing was perceived by their focus group participants to have improved mood and confidence among young people and young people preferred marketing contents on Facebook that seemed ‘user-generated’ or ‘subtle’ in appearance [33].

The qualitative studies reported that young people were skeptical towards the advertisements on sidebars (Facebook) or brand-sponsored advertisements [43,46] but they did not necessarily view their engagement with brand-related contents on SNS (i.e., “liking” or “sharing”) as a form of marketing, especially when these were shared by their peers [42,46,47]. Two studies also revealed the social acceptability to be seen consuming certain alcohol brands on SNS as these brands were linked to desired masculinity, femininity and cultural images [42,46].

Detrimental effects on young people’s intention to use the unhealthy commodities were demonstrated in seven out of the nine studies that included intended consumption as outcome measure [25,26,27,30,34,40,48] (Table 2). Among the studies that found significant detrimental associations, five studies were on alcohol [26,30,34,40,48], one on tobacco [27] and one on energy drinks [25]. Two studies did not find any significant association between digital marketing and consumption intention [36,45].

Detrimental effects on young people’s current use of unhealthy commodities were found in 11 out of the 17 studies that included current consumption as outcome measure (Table 2). Among the 11 studies that found significant detrimental association, eight were on alcohol [26,28,29,36,39,40,45,48], two were on tobacco [35,38] and one was on EDNP food [32]. Four studies found beneficial effects of digital marketing on current use of tobacco (*n* = 3) [27,31,49] and alcohol (*n* = 1) [30] products, meaning that exposure to digital marketing was associated with lower current use. Associations in two studies could not be concluded due to the very low reported digital marketing exposure and engagement that led to the exclusion of digital marketing variables in the regression models [41,50].

### Literature Appraisal

The majority of the studies were cross-sectional studies (*n* = 17) [26,27,28,29,30,31,32,33,37,38,39,40,41,45,47,48,49,50], the remainder were longitudinal studies (*n* = 2) [35,36], experimental studies, (*n* = 2) [25,34] and qualitative studies (*n* = 7) [42,43,44,46,47,51,52]. One qualitative study was reported as a short article and it was not possible to obtain further data, so this study was not appraised in this review [44].

In the study setup domain for critical appraisal, other than the longitudinal and qualitative studies, all other studies were rated as good other than one experimental study which did not describe randomization and concealment of treatment allocation [34]. Most of the included studies were rated as good in the sample selection domain, however 10 of the cross-sectional studies did not report the participation rate. In the assessment domain, the two experimental studies that objectively measured the study factors (i.e., exposure to digital marketing) were rated as good. The majority of the cross-sectional studies were rated as fair since most of the data were self-reported by the participants. Only one of the two longitudinal studies reported the follow-up rate (62%) [36]. Most of the qualitative studies were rated as good in the assessment domain by adopting at least two methodologies for data collection. Of note, researchers in two studies used “go-along interviews” and digital navigation software to store the participants’ online activities that included where they navigated through the sites [46,47]. In the data analysis domain, none of the cross-sectional studies justified their sample sizes. All studies controlled for potential covariates in their analyses including demographic variables, household and peers’ alcohol or tobacco use and psychological aspects such as sensation-seeking.

## 4. Discussion

This study systematically reviewed evidence regarding the marketing effects of unhealthy commodities promoted through digital platforms on young people’s attitudes and behaviors. The results indicated a link between digital marketing and young people’s attitudes towards and intended and current use of a range of unhealthy commodities including alcohol, tobacco and EDNP food or beverages. However, a definitive relationship could not be determined due to the heterogeneity of the study designs, study factors and outcome measures employed by the included studies. Our findings also suggested that the effects of digital marketing vary across the investigated commodities and according to the nature of the exposed digital marketing activities.

Overall, current evidence regarding the effects of digital marketing of unhealthy products highlighted its detrimental impacts, through improving attitudes towards (67% = 2/3 studies), enhancing intention to use (78% = 7/9 studies) and current use of (65% = 11/17 studies) these commodities. These effects seem to be the most consistent among studies on alcohol products, where five out of the seven studies found digital marketing increased intention to drink alcohol, while eight out of 10 studies found digital marketing increased actual alcohol consumption. Our findings are in accordance with the findings from an earlier systematic review that specifically focused on the impacts of digital alcohol marketing, which found that alcohol-related content on the Internet negatively influenced young people’s drinking behaviors [5].

The effects of digital marketing were reported to vary between products being advertised. While there was a fairly consistent association between digital alcohol marketing and young people’s behaviors, inconsistent findings were found in the tobacco studies. Among the five studies on tobacco products that investigated digital marketing impact on current tobacco use, two of them found non-smokers were more likely to notice tobacco marketing contents on the Internet [27,31], which was completely opposite to the other two tobacco studies that found smokers were more likely to be exposed to digital marketing of tobacco [35] and e-cigarettes [38]. One potential explanation for this could be the perceived images of these products among young people. The tobacco studies that found non-smokers were more likely to have reported seeing digital tobacco marketing were both conducted in Australia, where strong public health controls for tobacco products may have created unappealing images for these products [53,54] and people who disliked these products (non-smokers) might have paid more attention to the advertisements than current smokers. On the other hand, alcohol brands were marketed using themes of success, fun, masculinity and femininity; these were the images desired by and reported to be socially acceptable among young people to be seen with alcohol products on the SNS [42,46].

Differential impacts from the various digital marketing approaches were also noted. These marketing approaches were broadly categorized into three forms: (i) earned media where the marketing activities were peer endorsed (e.g., likes and comments on SNS by online communities); (ii) owned media where the marketing activities were generated by the company on the channels that it controlled (e.g., posts from company on their brand page); and (iii) paid media activities where the company paid advertisers to create marketing activities (e.g., display advertising) [55]. Significant detrimental effects were demonstrated in two studies [29,34] from the earned media activities but not the owned or paid media activities of digital marketing. An experimental study in which participants were exposed to Facebook marketing of a specific alcohol brand found that participants’ intended alcohol use was associated with the exposure to Facebook status updates (i.e., like, share, comment) (earned media). However, no significant effects on the intended alcohol use were found from the exposure to the online display advertisements on Facebook (paid media) [34]. Another study found participants’ engagement with Facebook alcohol marketing (liked, posted, commented or uploaded photos) (earned media) predicted alcohol use but no association was found with exposure to alcohol websites (owned media) [29].

Different impacts of the features (interactive and static) of the marketing activities can be explained by the findings from the included qualitative studies. Earned (interactive) media marketing activities, such as online peer networking especially through SNS, may blur the lines between commercial and user-generated content. It was reported in two studies that while participants denied having actively engaged with the digital marketing activities, many of them reported to have had shared amusing product-related contents with their peers on SNS [46,47]. The influential power of interactive marketing strategies, especially through social context endorsement (friends of endorsers on SNS or electronic word-of-mouth), has been well documented in the marketing research field. An experiment conducted to compare the effectiveness of various Facebook advertisements, including online banner advertisements and advertisements with the names of friends who were also fans on the Facebook page, revealed that the latter worked better in enhancing users’ impressions of the product [56]. It has also been revealed by advertising researchers that consumers are more likely to reject advertisements if marketers explicitly show their persuasive motives. However, these messages became more acceptable if their close acquaintances posted positive comments on the advertised product [56,57]. The seamless peer-to-peer transmission of marketers’ messages highlighted the challenges for the public health community to set boundaries and to safeguard young people from promotion of unhealthy products.

### 4.1. Strengths and Weaknesses of the Reviewed Studies

The reviewed studies were generally rated between fair and good. The majority of the studies were cross-sectional; the dearth of prospective longitudinal studies and controlled experimental studies limited the ability to make inferences on direction of causality for this research topic. Only one of the two longitudinal studies reported the follow-up participation rate and this study suffered systematic loss to follow-up (more than 20%) that may have introduced potential attrition bias on their results. More longitudinal studies or controlled experimental studies on this research topic appear warranted. The biggest weakness of the included studies was the self-reported method for capturing the exposure variables. The wide variation of the reported exposure and engagement rates discussed earlier could have resulted from the self-reported data.

### 4.2. Limitations and Future Research

Several limitations need to be considered when interpreting the results of this review. Firstly, there was a lack of standardization and consistency in measuring digital marketing exposure. The included studies examined marketing impacts of various features of marketing strategies, some more interactive than the others. Additionally, some studies examined mere exposure (if people had seen the promotion), while others examined engagement levels (e.g., likes, shares). The inconsistency of the examined study factors across the included studies may have led to varied study outcomes. Secondly, the majority of the studies included in this review were conducted in developed countries. Research in less economically developed countries is needed due to the growing unhealthy commodities promotion and the increased technology use in these countries [58,59]. Thirdly, digital marketing is only a small part of companies’ promotional efforts for their products. More weight can be added to the literature by comparing the influence of digital marketing to the marketing strategies carried out on different channels. Lastly, there is a possibility of publication bias that studies did not find any signification association may not have been published.

## 5. Conclusions

This review concludes that exposure to digital marketing may be associated with young people’s attitudes and behaviors for a range of unhealthy commodities. Marketing contents transmitted through young people’s social online interactions (earned media) blurs the boundary between user- and marketer-generated contents and appears to have a greater impact than the more explicit online advertisements (owned and paid media). Given the seamless and pervasive nature of the marketing activities on the digital platforms, there is a need for proactive consideration of effective regulation on unhealthy commodities marketed within the online environment. To our knowledge, this is the first review on the influence of this novel form of marketing exposure on young people. This review contributes to the small but growing body of evidence on this research topic by unravelling the complex relationship between marketing exposure and behaviors and identifying areas for future inquiry.

## Figures and Tables

**Table 1:** Search parameters for Systematic Review of the digital marketing effects on young people: Example on Scopus.

Operator	Definition	Hits
1. Title, Abstract, Keywords	market* OR advert* OR promot*	2,487,973
2. Title, Abstract, Keywords	online OR internet OR web OR “social media” OR “social network” OR “new media” OR “online game” OR advergam*	1,156,990
3. Title, Abstract, Keywords	“young people” OR “young adults” OR “young generation” OR “university students” OR “college students” OR adolescents OR teenagers OR youths	2,634,580
4. Title, Abstract, Keywords	child*	2,898,753
5. Title, Abstract, Keywords	food OR beverage OR drink OR soda OR cola OR alcohol OR tobacco OR cigarette	2,070,463
6. Boolean operator	#1 AND #2 AND #3 AND NOT #4 AND #5	931
7. Limit date range Limit language Limit document type	1990–2017EnglishArticle	780

**Table 2:** Characteristics and results of the included quantitative studies.

Author (Date)	Population (Country)	Study Aims	Data Collection (Study Design)	Study Factor	Outcome Measure	Results Control Variables (Overall Association)
Alhabash et al. (2015)	University students from introductory classes, mean age 21 years *n* = 379 (USA)	To examine the effects of viral behavioral intentions (intentions to like, share and comment on) for status updates and display advertisements on social media users’ intentions to consume alcohol	Experimental Design: 2 (likes: low vs. high) × 2 (shares: low vs. high) × (display ad type: alcohol ad vs. anti-binge drinking Public Service Announcement (PSA) vs. local bank) × 6 (status update repetitions) (Controlled intervention study)	Likes and shares on Facebook (Objectively measured)	Attitudes and viral behavioral intentions towards the display advertisements and status updates Intention to consume alcohol (Alcohol) (Self-reported)	Attitude towards status updates (*B* = 0.2, *t* = 4.5, *p* < 0.00) and viral behavioral intentions towards status updates (*B* = 0.5, *t* = 6.6, *p* < 0.00) positively predicted alcohol consumption intention. Attitudes towards ads display (*B* = −0.1, *t* = −1.6, ns) and viral behavioral intentions towards ads display (*B* = 0.1, *t* = 1.9, *p* = 0.06) did not predict alcohol consumption intention. No variables were adjusted. (Inconsistent association)
Buchanan et al. (2017)	Young adults aged 18–24 years *n* = 60 (Australia)	To assess the impact of online marketing on young adults’ perception and consumption behaviors, using energy drinks as an example	Pre-test/post-test experimental trial, followed by semi-structured interview (Controlled intervention study)	Experimental group: exposure to two energy drink brands website and social media sites (Objectively measured)	Attitudes towards, purchase intention and consumption intention of, the two exposed energy drinks brands and energy drinks products in general (Energy drinks) (Self-reported)	Exposure to energy drinks online marketing content improved young adults’ attitudes towards (*t*(50) = −4.5, *p* = 0.00) and increased consumption intention of (χ^2^(1) = 7.9, *p* = 0.01), energy drinks products. No variables were adjusted. (Significant detrimental association)
Carrotte et al. (2016)	Young people aged 15–29 years *n* = 1001 (Australia)	To explore the relationship between alcohol marketing on social media and alcohol consumption among young people	Online survey (Cross-sectional study)	Alcohol marketing social media use “like/follow pages on Facebook, Instagram or Twitter” (Self-reported)	Alcohol consumption (number of standard drinks consumed on a typical day of drinking and risky single occasion drinking) age of initiation of drinking (Alcohol) (Self-reported)	Liking or following any alcohol marketing page was significantly associated with early age (10–14 years) of first alcohol consumption (AOR = 2.2, 95% CI = 1.6–3.0). Higher AUDIT-C (more risky alcohol consumption) were associated with liking or following alcohol marketing pages (AOR = 2.1, 95% CI = 1.5–2.8). Adjusted variables: Gender, age, education, location, sexuality, country of birth, recreational spending per week, recent mental health problems, ever used illegal drugs, age at first alcohol consumption (Significant detrimental association)
Critchlow et al. (2016)	Young people aged 18–25 years *n* = 405 (UK)	To examine the relationship between awareness of traditional, digital marketing and young people’s frequency of high episodic drinking (HED)	Survey (Cross-sectional study)	Awareness of and participation with 11 digital marketing channels,’ awareness of nine traditional marketing channels (Self-reported)	Frequency of high episodic drinking (HED) (Alcohol) (Self-reported)	Participation with digital marketing increased the frequency of HED (*B* = 0.2, *p* < 0.00). Adjusted: Awareness of traditional alcohol marketing (Significant detrimental association)
De Bruijn et al. (2016)	European youths, mean age 14 years *n* = 9032 (Germany, Italy, Netherlands, Poland)	To examine the exposure to alcohol marketing through digital media and its association with initiation of alcohol use, recent binge drinking and volume of alcohol consumption	Survey (Cross-sectional study)	Frequency of exposure to alcohol marketing in online media. (Self-reported)	Alcohol use (Alcohol) (Self-reported)	Exposure to online alcohol marketing was linked to an increase likelihood of beginning alcohol use and binge drinking in the past 30 days. The association was the strongest for: looked at a website for alcohol brands (onset of drinking AOR = 1.1, 95% CI =1.1–1.2; past 30 days binge drinking AOR = 1.11, 95% CI = 1.1–1.2) downloaded alcohol-branded screensaver (onset of drinking AOR = 1.1, 95% CI = 1.1–1.2; past 30 days binge drinking AOR = 1.1, 95% CI = 1.1–1.2). Exposure to online alcohol ad increased the odds of being a drinker (AOR = 1.3, 95% CI = 1.2–1.4) and binge drinking (AOR = 1.24, 95% CI = 1.2–1.3) Adjusted: gender, smoking, age, education level, religious constraints against alcohol, alcohol use peers, alcohol use mother, peer permission to drink, maternal permission to drink. (Significant detrimental association)
Depue et al. (2015)	Connecticut residents aged 18–24 years *n* = 200 (USA)	To assess the association between smoking behavior and the exposure to mass media depictions of smoking on social networking websites	Telephone surveys (wave 1 and wave 2–5 months apart) (Longitudinal study)	See tobacco use on TV, in movies and in social media content such as Facebook or MySpace (Self-reported)	Cigarette use in the past 30 days (Tobacco) (Self-reported)	Time 1 social media tobacco use was a significant predictor of smoking at Time 2 (OR = 1.6, *p* < 0.05). Social media tobacco use had a moderate correlation to both time (*r* = 0.2, *p* < 0.05) and time 2 (*r* = 0.2, *p* < 0.05) Not adjusted: sex, race, friends and family tobacco use, sensation-seeking Social media depictions of tobacco use were associated with future smoking tendency (Significant detrimental association)
Dunlop et al. (2016)	Young people in two Australian states aged 12–24 years *n* = 8820 (Australia)	To assess the exposure of young Australians to online tobacco advertising and promotion and to determine whether exposure has changed in recent year in relation to the changes in tobacco promotion opportunities	Telephone surveys (four waves) (Repeat cross-sectional study)	Exposure to Internet-based tobacco advertising and branding in the past month (Self-reported)	Smoking behaviors: Current smoking (never-smokers; experimenters; current smokers; ex-smokers), smoking susceptibility (Tobacco) (Self-reported)	Current or ex-smokers had lower odds of being exposed to Internet-based advertising than experimenters or never-smokers (AOR = 0.4, 95% CI = 0.3–0.5) Non-smokers aged 12–17 years, exposure to online advertising and branding (OR = 1.3, 95% CI = 1.1–1.6) or branding alone (OR = 1.4, 95% CI = 1.1–1.8) increased their susceptibility to smoking Adjusted: demographic characteristics, year of Interview, average daily Internet use, SES status, smoking exposures (friends, household) (Inconsistent association)
Gordon et al. (2011)	Students attending schools in the West of Scotland, aged 12–14 years *n* = 920 (UK)	To examine the cumulative impact of alcohol marketing communications on adolescents’ drinking behaviors	Survey (Cross-sectional study)	Awareness, appreciation and involvement with various forms of alcohol marketing including digital marketing, as measured by interview-administered questionnaire (Self-reported)	Drinking status, future drinking intentions, age of initiation of drinking, as measured by self-completion questionnaire (Alcohol) (Self-reported)	Participation in electronic alcohol marketing increased the likelihood of being a drinker (OR = 4.0, 95% CI = 1.5–10.8) and associated with greater intention to drink alcohol in the next year (*B* = 0.1, *p* < 0.01) Adjusted: perceived parental attitudes towards drinking and alcohol consumptions, perceived siblings and peers’ attitudes towards drinking and alcohol consumption, liking of adverts in general and liking of alcohol adverts in particular, age (Significant detrimental association)
Hoffman et al. (2014)	Public and private university students, mean age 21.4 years *n* = 637 (USA)	To examine the relationship between college students’ use of social media, their exposure to alcohol marketing messages through social media and their alcohol-related beliefs and behaviors	Online survey (Cross-sectional study)	Engage with alcohol related marketing on the websites and social media sites. (Self-reported)	Drinking behaviors: problem drinking as measured by problem-drinking index, use in past 30 days, use in 1 occasion. (Alcohol) (Self-reported)	The use of alcohol-marketing applications on social media predicted: more drinking problems (*B* = 0.3, *p* < 0.00), more frequent alcohol use in past 30 days (*B* = 0.2, *p* < 0.00), heavier consumption in a single occasion (*B* = 0.2, *p* < 0.00) Adjusted: private or public university affiliation, demographic variables included sex, age, reported family income, reported grades in school, expectations for educational attainment, year in college (Significant detrimental association)
Jones and Magee (2011)	Adolescents aged 12–17 years *n* = 1113 (Australia)	To investigate the exposure level to different types of alcohol advertising and to examine the association between exposure to advertising and alcohol consumption	Survey (Cross-sectional study)	Exposure to alcohol advertisement across eight media including Internet (Self-reported)	Alcohol consumption behaviors (initiation, recent consumption in the past 4 weeks and frequency of consumption in the previous 12 months) (Alcohol) (Self-reported)	Exposure to Internet alcohol advertising increased the likelihood of recent alcohol consumption (AOR = 1.4, 95% CI = 1.0–1.8) but not the alcohol initiation (AOR = 1.3, 95% CI = 0.9–1.7) or alcohol consumption in the past 12 months (AOR = 1.0, 95% CI = 0.7–1.3) Adjusted: age, gender, country of birth, religion, mother’s alcohol consumption, father’s alcohol consumption, siblings’ alcohol consumption, friends’ alcohol consumption, source of recruitment. (Inconsistent association)
Jones et al. (2016)	Young people aged 16–24 years *n* = 283 (Australia)	To examine the association between Facebook users’ interactions with alcohol brands and alcohol consumption	Online survey (Cross-sectional study)	Recalled exposure to alcohol marketing on Facebook, interaction with alcohol brands on Facebook (e.g., liking, commenting) (Self-reported)	Alcohol use amount (1–2 drinks, 3–4 drinks and more than 5 drinks), alcohol use frequency, binge drinking frequency as measured by AUDIT-C. (Alcohol) (Self-reported)	Respondents who had ever liked, posted, commented or uploaded/tagged alcohol brands on Facebook increased the alcohol use frequency (OR = 2.0, 95% CI = 1.2–3.5); increased alcohol amount use (OR = 3.7, 95% CI = 2.1–6.7), increased binge drinking frequency (OR = 2.4, 95% CI = 1.4–4.2) No association was found between the quantity of alcohol consumed and having visited an alcohol’s Facebook page, visited an alcohol website by clicking the link on Facebook, or viewed an event created/sponsored by an alcohol company Adjusted: socio-demographic backgrounds (Inconsistent association)
Lin et al. (2012)	Students aged 13–14 years *n* = 2538 (New Zealand)	To examine to association between awareness and engagement with a range of alcohol marketing channels and drinking behaviors	Computer assisted telephone interview (Cross-sectional study)	Awareness of and engagement with 15 of alcohol marketing channels including web based marketing, as measured by interview-administered questionnaire (Self-reported)	Drinking status, drinking frequency, drinking quantity and future drinking intentions, as measured by interview-administered questionnaire (Alcohol) (Self-reported)	Those engaged with web-based alcohol marketing were: More likely to be drinkers (OR = 1.9, 95% CI = 1.2–3.0) More likely to have drunk alcohol in the past 12 months (OR = 2.0, 95% CI = 1.2–3.2), Less likely to drink alcohol on a typical occasion (OR = 0.7, 95% CI = 0.5–1.0) Not significantly related to drinking intention (OR = 1.0, 95% CI = 0.4–2.2) or drinking frequency (OR = 0.9, 95% CI = 0.6–1.2) Adjusted: age, gender, ethnicity, drinking behaviors and perceived drinking approval of parents, siblings and friends (Inconsistent association)
MacFadyen et al. (2001)	Young people aged 15 and 16 years *n* = 629 (UK)	To examine the association between young people’s awareness of and involvement with tobacco marketing and their smoking behavior	Survey (Cross-sectional study)	Exposure and involvement to all forms of tobacco marketing activities including Internet sites (Self-reported)	Smoking status (non-smoker; tried smoking; current smoker) (Tobacco) (Self-reported)	There was a low number of participants (8%) who were aware of the Internet sites for cigarettes or smoking and their smoking status were not significantly different (*p* = 0.36). Digital marketing exposure and involvement variables were not included in the regression models. Adjusted: gender, age, friends’ smoking, sibling’s smoking, mother’s smoking and father’s smoking, socioeconomic group, marital status of parents, future education intentions and parental presence during interviews (Association cannot be determined)
McClure et al. (2016)	Youths aged 15–20 years *n* = 2012 (USA)	To examine the longitudinal association between Internet alcohol marketing engagement and alcohol use transitions among youth	Surveys were conducted at two time points (1 year apart) (Longitudinal study)	Internet alcohol marketing receptivity: exposure to alcohol advertising on the Internet, visiting alcohol brand websites, being an online alcohol brand fan (Self-reported)	Ever drinking and binge drinking (6 or more drinks per occasion) (Alcohol) (Self-reported)	Internet alcohol marketing receptivity increased the likelihood of initiating binge drinking, the higher the receptivity score, the greater the impact (score 1: OR = 1.8, 95% CI = 1.1–2.8; score 2: OR = 2.2, 95% CI = 1.1–4.4) However, Internet alcohol marketing was not associated with the initiation of ever drinking (score 1: OR = 1.2, 95% CI = 0.8–1.9; score 2: OR = 1.1, 95% CI = 0.3–3.8, ns) Adjusted: baseline drinking status, socio-demographics, peer drinking, parent drinking, general time spent on the Internet, sensation seeking (Inconsistent association)
Perez et al. (2012)	Adolescents and young adults aged 12 to 24 years *n* = 1000 (Australia)	To examine the level of exposure of New South Wales (NSW) adolescents and young adults to the promotion of tobacco through point-of-sale, Internet, entertainment media and venues and to identify young people who are at risk of exposure	Telephone survey (Cross-sectional study)	Perceived exposure to promotion or advertising of tobacco in the last month through various forms of marketing methods including Internet (Self-reported)	Smoking status (current smokers, ex-smokers, experimenters, non-smokers) and susceptibility to smoking (susceptible non-smokers, non-susceptible non-smokers) (Tobacco) (Self-reported)	Participants who had ever smoke had lower odds of seeing cigarette brands, tobacco company names or logos on the Internet (OR = 0.6, 95% CI = 0.4–1.0) than those who never smoke. Adjusted: age, sex, Socio-economic status (SES), income, household smoking, friends smoking, Internet use (Significant beneficial association)
Pinsky et al. (2010)	Subjects aged 14–25 years *n*= 1091 (Brazil)	To explore Brazilian adolescents and young adults’ exposure to alcohol advertising and to assess the relationship between the exposure to heavy alcohol consumption	Face-to-face interviews but quantitative questions (Cross-sectional study)	Perceived exposure to alcohol marketing in different media including Internet (Self-reported)	Alcohol consumption: high intensity drinkers (drink at least once a week) vs. low intensity drinkers (drink less than once a week) (Alcohol) (Self-reported)	91.6% declared they have not seen alcohol advertising on the Internet or visited a website related to alcohol beverages. Exposure to alcohol Internet sites was not included in the logistic models, due to low incidence of reported exposure Adjusted: intensity of alcohol consumption, sociodemographic backgrounds (Association cannot be determined)
Reinhold et al. (2017)	Students at a large Midwestern university aged 18–24 years *n* = 5983 (USA)	To explore young adults’ perceptions of harm and acceptability of the use of e-cigarette and to examine whether e-cigarette advertising has an effect on perception of harm and acceptability of use	Online survey (Cross-sectional study)	E-cigarette advertising exposure through different media channels including Internet (Self-reported)	Lifetime e-cigarette use, perception of harm, addictiveness and acceptability of e-cigarette use in places (E-cigarette) (Self-reported)	Having seen an advertisement on the Internet was significantly associated with lower perceived harm of e-cigarette use (AOR = 1.2, 95% CI = 1.1–1.3) and also acceptability of e-cigarette use in various locations (all *p* < 0.00) Having seen advertisement on the Internet was not associated with the lower perceived addictiveness of e-cigarette (AOR = 1.1, 95% CI = 1.0–1.2, ns) Adjusted: maternal smoking status, smoking history, gender, race, exposure to advertising on other platforms (TV, magazine) (Inconsistent association)
Salgado et al. (2014)	Current or recently graduated medical students aged 20–30 years *n* = 1659 (Argentina)	To examine the effects of tobacco industry Internet marketing strategies on young adults	Survey (Cross-sectional study)	Frequency of access to tobacco website (from “once a day or more” to “once a month or less”). (Self-reported)	Ever smoke, never smoke, current smoker, former smoker (Tobacco) (Self-reported)	Former or current smokers were more likely to have accessed a tobacco brand website at least once (AOR = 2.5, 95% CI = 1.4–4.2; AOR = 8.1, 95% CI = 4.7–14.2, respectively) Current smokers were less likely to report having seen a tobacco advertisement on the Internet (AOR = 0.6, 95%CI = 0.5–0.8) Adjusted: age, daily use of Internet, received tobacco marketing promotion, used tobacco marketing promotion (Inconsistent association)
Scully et al. (2012)	Secondary students aged 12–17 years *n* = 12,188 (Australia)	To determine the associations between exposure to various types of food marketing and adolescents’ food choices and food consumption	Online survey (Cross-sectional study)	Various types of food marketing exposure including Internet (Self-reported)	Food choices, eating behaviors- frequency of consumption of fast food, sugary drinks and sweet snacks (Energy-dense and nutrient poor (EDNP) foods) (Self-reported)	Exposure to the digital food marketing increased the odds: To consume fast food one exposure source (OR = 1.2, 95% CI = 1.1–1.4) two exposure sources (OR = 2.3, 95% CI = 1.9–2.7) To consume sugary drinks two exposure sources (OR = 1.3, 95% CI = 1.1–1.6) To consume salty snacks two exposure sources (OR = 1.3, 95% CI = 1.1–1.5) Adjusted: gender, school year, geographic location of residence, socio-economic position (SEP), body mass index (BMI), school level (Significant detrimental association)
Singh et al. (2016)	Middle and high school students grades 6 to 12 (12–18 years) *n* = 22007 (USA)	To examine the association between e-cigarette advertising exposure (four sorts including Internet) and current e-cigarette use among US youth	Survey (Cross-sectional study)	Exposure to e-cigarette advertisement on Internet, newspaper/magazines, in retail stores, in TV/movies (Self-reported)	Current cigarette use (in the past 30 days) (E-cigarette) (Self-reported)	Among middle school students, greater exposure to e-cigarette Internet advertising increased the odds of being current e-cigarette users (most of the time/always AOR = 2.9, 95% CI = 1.9–4.5) Among high school students, greater exposure to e-cigarette Internet advertising increased the odds of being current e-cigarette users (most of the time/always AOR = 2.0, 95% CI = 1.7–2.5) Adjusted: gender, ethnicity, grade, other tobacco use (Significant detrimental association)
Weaver et al. (2016)	Young people aged 16–29 years *n* = 172 (Australia)	To investigate young people’s perception of alcohol advertising on Facebook and to investigate the perceived compliance of these advertising with the Alcohol Beverages Advertising Code (ABAC)	Focused group discussion (to inform development of online survey) Online survey (Cross-sectional study)	Exposed to six popular Australian alcohol brands’ Facebook pages (Self-reported)	Perception and interpretation of specific alcohol-branded marketing on Facebook, as measured by open-ended questions (with and without prompts). Drinking behaviors (Alcohol) (Self-reported—a mixture of quantitative and qualitative findings)	The focused group discussion revealed that participants preferred alcohol advertising that was ‘user-generated’, ‘casual’ and ‘subtle’ in appearance as it gives the impression that it was created by a ‘real person’ Association with success was also the most frequently reported message, for example, ‘drinking is a social event and aids in the betterment of your social status’ With prompts, participants reported that alcohol advertising made them feel more relax (67%), improved mood (65%), feel more social and outgoing (57%) and confident (49%) Measured but not adjusted: age, sex, education levels, favorite type of alcohol (Association cannot be determined)

PSA: Public Service Announcement; *B*: Standardized regression coefficients; *p*: Level of marginal significance; AOR: Adjusted odds ratio; CI: Confidence Interval; SES: Socio-economic status.

**Table 3:** Characteristics and results of the included qualitative studies.

No.	Author (Date)	Population (Country)	Study Aim (Product)	Data Collection	Results
1	Atkinson et al. (2017)	Young people aged 16–21 years *n* = 70 (UK)	To analyze the use and contents of alcohol marketing on the social network sites (SNS) and to explore young people’s perspectives and experiences on alcohol marketing on SNS (Alcohol)	Stage 1: Content analysis of five alcohol brands’ interaction with users on social networking sites; both brand- and user-generated contents over 1-month period Stage 2: Fourteen semi-structured interviews with peer groups of young people	Alcohol industry used social networking site particularly Facebook to engage consumers Branding of alcohol appealed young people. The social acceptability of consuming certain drinks and brands and being ‘seen’ drinking these on SNS were influenced by the connotations of masculinity, femininity and maturity Influence of SNS marketing on young people was mediated through their peers’ online activities- engagement with alcohol on SNS reported to be done through young people’s news feed as a result of their friend’s interaction or through third party content (e.g., music and sporting events)
2	Gaber and Wright (2014)	Young people aged 17–29 years *n* = 40 (Egypt)	To explore the factors that influence young Egyptians’ attitudes towards fast-food advertising on Facebook. (Fast-food)	Focus groups Content analysis	Most of the participants had positive attitudes towards the advertising on Facebook and believed that Facebook advertising is informative and credible Participants preferred Facebook advertising over web advertisements that appear pop up causing a big amount of inconvenience and interruption Having friends who also liked or commented on the Fast food Facebook pages increased the likelihood of consumers clicked on the advertisement or tried the brands
3	Lyons et al. (2015)	18–25 years old young people *n* = 141 (focus group discussion) *n* = 23 (individual interviews) (New Zealand)	To use an innovative qualitative methodology to explore the role of social networking site in drinking cultures and alcohol consumption practices among young adults(Alcohol)	Stage 1: Focus group discussion Stage 2: Individual interviews with Internet-enabled laptop (digital navigation software to store all online activities) Stage 3: Analysis of a database of web-based materials that were mentioned or shown by participants in Stage 1 and 2	Alcohol companies use social media to enhance identity displays; participants actively engaged with these marketing initiatives with many highlighted that alcohol brands and pages were integral part of their online identities; allowed them to present their tastes and preferences and socially interacted with the other Facebook users by sharing amusing alcohol-related content generated by alcohol companies Participants do not necessarily view alcohol product pages and promotions on Facebook as advertising; alcohol marketing on Facebook involved Facebook friend relationships, that is, appear in group links, news feeds and status updates which are the in the same manner as friends’ postings
4	Moraes et al. (2014)	Young adults aged 18 to 24 years *n* = 15 (UK)	To explore the use of Facebook to promote alcohol use among young people (Alcohol)	Focus group Netnographic study (apply the ethnographic research methods to study the cultures and communities that emerged through computer-mediated communications)	Facebook was used as a tool by alcohol brands and nightclub to communicate, co-produce and co-generate alcohol-related contents with young people that encourages alcohol use Wall comments, drinking-related group memberships, events, photographs and other social communications on Facebook normalized alcohol consumption among young people The events application was identified as one of the most valued Facebook features. For instance, by sending emails to users through events section, Vodka-Energy not only advertised their parties, they also promoted their sites and alcohol deals
5	Niland et al. (2017)	Young adults aged 18–25 years *n* = 7 (New Zealand)	To examine young adults’ interactions with alcohol marketing from within their own social networking practices and to examine participants’ meanings and understandings of the ways in which commercial alcohol interests interacted with their own online practices. (Alcohol)	Go-along interviews- participants accessed and navigated through their Facebook accounts and took the researcher on a “tour” showing and elaborating their social networking practices (data screen- capture software to track participants’ online navigation and audio-visual recording of the conversation and non-verbal behaviors)	All participants viewed Facebook advertising as the sponsored sidebar ads on their Newsfeed pages, participants did not interpret ‘liking’ alcohol-related content or alcohol venue page photos and activities as a form of marketing Alcohol online marketing embedded in friendship endorsements and invitations makes the presence of Facebook alcohol marketing obscured since it was simply part of routine online friendship activities. Alcohol marketing on venue pages was not viewed as alcohol marketing but as prompts for friends to drink together Online marketing was explicitly employed by participants as funny user-generated content to share with friends instead of marketing contents
6	Purves et al. (2015)	14–17 years young people *n* = 48 (UK)	To explore the ways that alcohol marketers engage with consumers on the social media sites (Alcohol)	Content analysis by netnographic approaches Focus groups (single sex friendship groups)	Brand communicates their personality through social networking sites. Brand preference indicated the characteristics of young people. For example, males and females may prefer different alcohol brands Participants in the focus group reported seeing large volume of alcohol products marketing on the social networking sites and these were viewed as an inevitable daily content of social networking sites. Participants also reported to be exposed to these marketing contents due to their friends ‘liked’ or ‘re-tweeted’ posts from alcohol brands
7	Waqa et al. (2015)	Students aged 14–17 years *n* = 30 (Fiji)	To explore Fijian students’ view on tobacco and tobacco-related media depictions to gain insight into the drivers of smoking uptake and for potential direction for prevention intervention. (Tobacco)	In-depth interviews	Internet was identified by the young Fijians as an important source of information about tobacco promotion that persuade young people to smoke via repeat screenings and interactive applications and platforms Tobacco related media depictions on the Internet for example celebrity smoking images was viewed by participants as sending the negative messages to young people. Media linked tobacco use to “becoming famous”
